# Orthotopic Liver Transplantation for Etanercept-induced Acute Hepatic Failure: A Case Report

**DOI:** 10.30699/ijp.2020.117000.2269

**Published:** 2020-07-16

**Authors:** Dorsay Sadeghian, Farid Azmoudeh-Ardalan, Simin Dashti-Khavidaki, Nasir Fakhar

**Affiliations:** 1 *Department of Pathology, Tehran University of Medical Sciences, Tehran, Iran*; 2 *Department of Clinical Pharmacy, Faculty of Pharmacy, Tehran University of Medical Sciences, Tehran, Iran*; 3 *Liver Transplantation Research Center, Tehran University of Medical Sciences, Tehran, Iran*

**Keywords:** Autoimmunity, Etanercept, Hepatitis, Hepatic failure, Liver transplantation

## Abstract

Occurrence of hepatotoxicity following prescription of etanercept, a tumor necrosis factor-alpha (TNF-α) antagonist, is a relatively well-known issue. On the other hand, acute hepatic failure which could lead to liver transplantation is extremely rare to the best of our knowledge, and there is no previously published case in the literature. In this article, we presented a case of acute liver failure followed by liver transplantaion, in a 32- year old man with a previous history of ankylosing spondylitis after etanercept usage. On pathologic examination of the explanted liver of the patient, extensive confluent necrosis in all liver segments with prominent infiltration of a mixed population of inflammatory cells in portal tracts was noticed. In conclusion, close follow-up of patients who are receiving etanercept is crucial, since its liver complications could be severe enough to subject the patients for liver transplantation.

## Introduction

From 1998 to 2009, tumor necrosis factor-alpha (TNF-α) antagonists including infliximab, etanercept, adalimumab, certolizumab, and golimumab were approved for treating several inflammatory diseases. Although drug-induced liver injury (DILI) was not reported during phase III clinical trials, post-marketing surveillance showed DILI by all TNF-α antagonists (1). It seems that among older and more widely used TNF-α antagonists (infliximab, Etanercept, and adalimumab), the most DILI cases have been reported by infliximab although not more commonly used than Etanercept and adalimumab (1-4). Some reported cases proposed no cross hepatotoxicity between infliximab and etanercept (5,6) or between adalimumab and etanercept (7) showing that a “class effect” is not universal among anti-TNF-α agents (1). 

While all types of liver damage including hepatocellular and cholestatic types have been reported, the most reported types of hepatotoxicity by TNF-α antagonists is hepatocellular injury with features of autoimmunity as shown by histopathology of liver biopsy and serologic findings of high titers of anti-nuclear antibodies (ANA), anti-smooth muscle antibodies (ASMA), and/or elevated immunoglobulin G (1,8). The median times from initiation of anti-TNF-α agents and DILI development differ from 14 to 18 weeks with time ranges of DILI happening from 2 to 104 weeks in different reviews of reported cases (2-4).

Although several cases of Etanercept-induced hepatotoxicity have been reported, to our knowledge, a tcase of Etanercept-induced acute hepatic failure which necessitates liver transplantation, has not been reported in the literature.

##  Case Report

Our patient was a 32-year-old man, known case of ankylosing spondylitis with positive HLA-B27, diagnosed on september, 2016. He initially was treated with corticosteroids, sulfasalazine, and indomethacin, for several months. Due to the incomplete therapeutic response, after complete routine laboratory tests, latent tuberculosis and viral infections work-up, his regimen was changed to anti-TNF-α (Etanercept) with a dosage of 25 mg weekly and after that increased to 50 mg per week from September 15^th^, 2016. After a month his symptoms were significantly improved. Five months later, on February 12^th^, 2017, the patient presented with raised liver transaminases (AST:300 IU/L and ALT:100 IU/L) and poly gammopathy, which were detected in his routine 

**Fig. 1:**
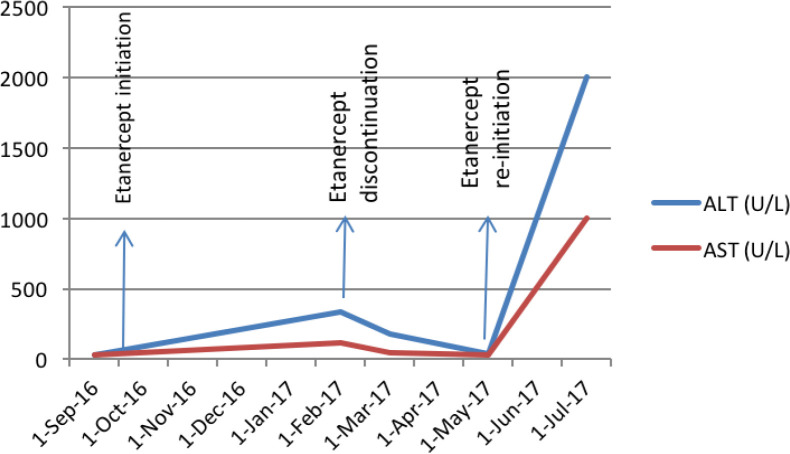
Patient's serum transaminases level during Etanercept treatment

**Fig 2:**
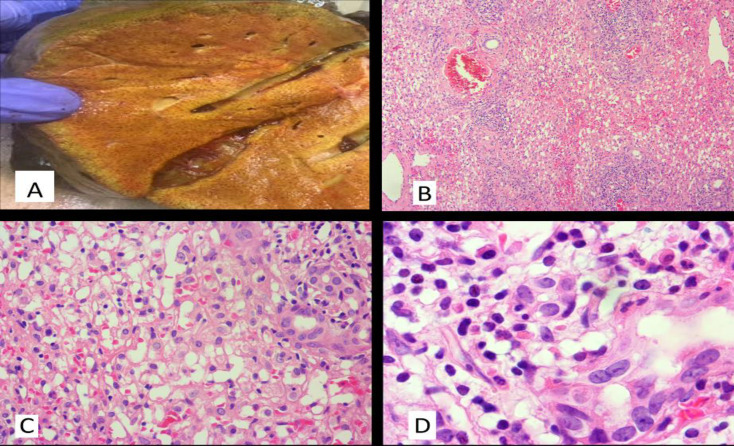
A. Mottled cut surface of the liver in gross examination. B. Confluent necrosis in the liver parenchyma (x100). C. Mild cholangiolar reaction as well as infiltration of mix population inflammatory cells is noted in portal tracts (x400). D. Prominent eosinophilic infiltration in the portal tracts (x1000)

follow-up laboratory tests without any obvious clinical symptoms. Abdominal and pelvic ultrasounds had normal findings. He also used prednisolone, silymarin, alendronate and calcium, and vitamin D supplements. In this setting, etanercept was discontinued for the patient. After 2 months, his liver enzymes returned to the normal level, so the anti-TNF-α regimen started again with the same dosage as the beginning on May 13^th^, 2017. After 2 months in July 2017, the patient was admitted to the emergency department of Tehran Imam-Khomeini Hospital Complex with icterus, fatigue, and weakness. He was completely alert initially. His laboratory tests showed AST: 2579 IU/L, ALT:2854 IU/L, ALP:648 IU/L, INR:5.9, Bilirubin total: 15.4 mg/dl and direct: 10.5 mg/dl. Figure 1 shows his serum transaminases during etanercept treatment and discontinuation phases. His serologic markers had negative results for hepatitis B, hepatitis C, human immunodeficiency viruses or cytomegalovirus infections. He was not a smoker and denied alcohol use. The patient weight was 76 Kg (BMI=25.39kg/m^2^). In the next two weeks, his liver enzymes did not change significantly, also his level of consciousness dramatically decreased and after 15 days on July 27^th^, 2017, a grade II liver encephalopathy occurred. Therefore, he received emergent liver transplantation from a deceased donor with the clinical impression of fulminant hepatitis.

Etanercept induced DILI was highly probable (score 10) according to Council for International Organization Medical Sciences/Roussel Uclaf Causality Assessment Method (CIOMS/RUCAM) scale (9) and certainly according to the World Health Organization-Uppsala Monitoring Center (WHO-UMC) causality assessment (10) in this patient.

**Pathologic Findings:**

The explanted liver of the patient was received in the pathology department. The specimen was composed of a total liver with an attached gallbladder, weighing 950g and measuring 20 x14 x 8 cm. Cut sections through the liver parenchyma showed a mottled surface with no obvious prominent nodule or mass lesion (Figure 2A). Tissue samples were obtained from all 8 liver segments. The microscopic examination of prepared sections showed extensive confluent necrosis in all liver segments. In portal tracts, prominent infiltration of a mixed population of inflammatory cells composed of lymphoplasma cells, eosinophils and neutrophils were noticed. Also, a moderate to severe cholangiolar reaction was prominent (Figure 2B-D)**. **No significant fibrosis was identified on trichrome staining.

## Discussion

Hepatotoxicity has been reported by all TNF-α antagonists including etanercept (1-4). The most common type of etanercept-induced DILI is the hepatocellular pattern of injury, sometimes with features of autoimmune hepatitis (1,11). Increase in serum ALT is usually mild (11). A case of granulomatous hepatitis has been reported with etanercept in a patient with rheumatoid arthritis (12). In these cases, liver enzymes returned to normal range 2-11 weeks after etanercept discontinuation in different cases (12,13). In some cases, etanercept treatment reinitiated with lower doses (12-15) without an increase in liver enzymes and few cases were treated with changing to another TNF-α antagonist (16).

Van Denderen *et al.* reported a possible or probable etanercept-induced liver dysfunction in 9 of 105 (approximately 9%) patients with ankylosing spondylitis (17) that is higher than the incidence of 1-1.1% reported in patients with rheumatoid arthritis (18). In all but one case reported by Van Denderen *et al.* liver enzymes increased 1 to 3 months after etanercept initiation. In one patient hepatotoxicity happened after 6 months. No differences in age, sex or alcohol consumption were reported between etanercept treated patients with and without hepatotoxicity. Patients with a higher body mass index had a higher risk of etanercept induced hepatotoxicity (*P*<0.001) (17). Some authors suggested the possibility of more frequency of anti-TNF alpha-induced autoimmune hepatitis in patients with ankylosing spondylitis compared to patients with rheumatoid arthritis because of less immunosuppression therapy in these patients (19). Our patient had ankylosing spondylitis as well but did not have a high body mass index. He showed hepatocellular liver injury based on the patterns of changes in serum enzymes and histopathological study of the explanted liver. He also had some features of autoimmunity such as positive serum anti-nuclear antibody (ANA) and polygammopathy. He underwent a liver transplnatation because of life-threatening liver failure and hepatic encephalopathy.

## Conclusion

Although a severe hepatic failure, liver transplantation, and death have been reported by infliximab, to our knowledge, the presented case is the first case of etanerceptinduced severe hepatic damage that indicates a liver transplantation. In conclusion, a careful monitor is necessary for those patients who are candidate for etanercept treatment. 
